# THz-photonics transceivers by all-dielectric phonon-polariton nonlinear nanoantennas

**DOI:** 10.1038/s41598-022-08695-y

**Published:** 2022-03-17

**Authors:** Unai Arregui Leon, Davide Rocco, Luca Carletti, Marco Peccianti, Stefano Maci, Giuseppe Della Valle, Costantino De Angelis

**Affiliations:** 1 Department of Physics, Politecnico di Milano, Piazza Leonardo da Vinci 32, 20133 Milan, Italy; 2 Department of Information Engineering, University of Brescia, via Branze 38, 25123 Brescia, Italy; 3 National Institute of Optics, Consiglio Nazionale delle Ricerche, via Branze 45, 25123 Brescia, Italy; 4 Emergent Photonics Lab (EPic), Department of Physics and Astronomy, University of Sussex, Brighton, BN1 9QH UK; 5 Department of Information Engineering and Mathematics, University of Siena, 53100 Siena, Italy; 6 Institute for Photonics and Nanotechnologies, Consiglio Nazionale delle Ricerche, Piazza Leonardo da Vinci 32, Milan, 20133 Italy

**Keywords:** Metamaterials, Nanocavities, Nanoparticles

## Abstract

The THz spectrum (spanning from 0.3 to 30 THz) offers the potential of a plethora of applications, ranging from the imaging through non transparent media to wireless-over-fiber communications and THz-photonics. The latter framework would greatly benefit from the development of optical-to-THz wavelength converters. Exploiting Difference Frequency Generation in a nonlinear all dielectric nanoantenna, we propose a compact solution to this problem. By means of a near-infrared pump beam (at $\omega _1$), the information signal in the optical domain (at $\omega _2$) is converted to the THz band (at $\omega _3=\omega _2-\omega _1$). The approach is completely transparent with respect to the modulation format, and can be easily integrated in a metasurface platform for simultaneous frequency and spatial moulding of THz beams.

## Introduction

The terahertz (THz) region of the electromagnetic spectrum has gained increasing attention in the last decades and today it is one of the fundamental emerging branches of the broad photonics community. Astrophysics, communications, imaging, spectroscopy, biotechnology and security are among the huge plethora of applications where THz technology can provide groundbreaking devices and systems, also thanks to the particular features of a wide range of media in the THz spectral range^1–9^. Research is thus today focused on overcoming or bypassing difficulties in the advancement of this technology, such as the scarcity of transmitters and receivers and the sometimes poor availability of components able to manipulate THz radiation (lenses, polarizers, mirrors, beam splitters, etc.).

**Figure 1:**
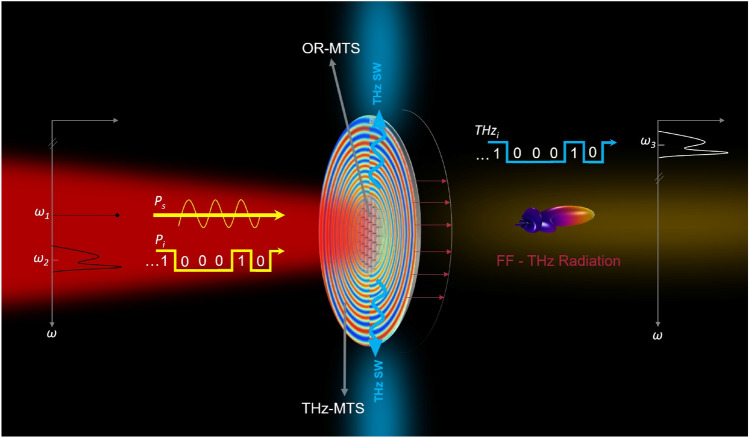
The wavelength converter: the information message, with power $P_i$, mixes with the incident signal, power $P_s$, both in the IR region. DFG in the all–dielectric nanoantennas emitting the THz information signal, $THz_i$, to produce a THz surface wave (SW) which propagates in the surrounding THz metasurface where it is converted into the desired THz radiation in the far-field (FF). The FF plot is created by using COMSOL Multiphysics v5.5—https://www.comsol.com.

Regarding THz beam control, phased array devices, leaky-wave antennas, reflectarrays, and transmitarrays can often be suitable for ad hoc wavefront engineering. Noteworthy, metasurfaces are today identified as the common framework where all the above approaches can be conveniently unified^10^ to provide a compact and integrable solution to a variety of different beam forming needs. For example, in Fig. 1 the THz surface wave is beam formed into the far-field (FF) THz radiation by a modulated THz metasurface, providing gain and directionality as suggested in^11^.

As far as transmitters are concerned, modulated THz generation is needed with high repetition rate in different spectral regions. Despite the fact that THz sources are still lacking in some spectral regions, photoinduced THz generation is today obtained by various mechanisms^12–17^. The most frequently addressed approaches in the extensive existing literature consist of THz-Quantum Cascade Lasers (THz-QCLs) and photoconductive antennas (PCAs); the latter are able to reach, in pulsed regime, average THz output powers of up to few hundreds of microwatts via optimization with plasmonic electrodes^18^, while THz-QCLs can provide peak THz output powers exceeding 1 W^19^. These solutions, though, involve large generating volumes, operate at frequencies roughly below 5 THz and are particularly suitable for spectroscopic or imaging applications. Recently, in order to get the highest performance per unit volume via resonant, nanoscale, compact platforms, THz generation by optical rectification in plasmonic Split-Ring Resonators (SRRs) has been reported^20,21^; the THz signal is produced from nanoscale SRRs by exciting magnetic-dipole modes in the infrared regime. A thorough study of THz pulse generation using a 40 nm thin metasurface based on plasmonic SRRs excited with a laser oscillator emitting nanojoule femtosecond pulses has been reported in^22^, measuring a conversion efficiency as high as that of a 0.1 mm thick ZnTe crystal. Despite these pioneering results, a breakthrough in the field demands for a substantial improvement of the conversion efficiency at the nanoscale.

One promising alternative to plasmonic THz emitters is represented by all-dielectric THz antennas. Such a structure has already gained a lot of attention in nonlinear optics as a building block for second and third harmonic generation in dielectric metasurfaces^23–26^.

Noteworthy, Difference Frequency Generation (DFG) in both plasmonic and all-dielectric nananoantennas is also attractive because it offers the possibility of down converting into the THz region an information signal which is generated and modulated in the telecommunication window where a complete palette of devices is already available. As schematically depicted in Fig. 1, exploiting DFG driven by second-order nonlinearities in a nonlinear all dielectric nanoantenna, we propose here this wavelength converter. By means of a near-IR pump beam (at $\omega _1$), the information signal in the optical domain (at $\omega _2$) is converted to the THz band (at $\omega _3=\omega _2-\omega _1$). Remarkably, by changing the frequency of the pump beam we can span the entire THz spectral band, provided the material platform is suitable. Importantly, since the information is directly converted into the THz radiated light, the proposed mechanism of IR to THz frequency conversion does not required any additional component: it is therefore transparent with respect to the modulation scheme and format of the information signal. As such, it can be easily integrated in a metasurface platform, paving the way to the development of all-optical ultracompact devices for simultaneous frequency and spatial moulding of THz beams. Furthermore, the reciprocal THz-to-optical up-conversion can be similarly held in the proposed THz-photonics structure, which subsequently acquires the potential to perform as an efficient miniaturized transceiver for wireless communication applications^27–29^.

As a prototype example, for an AlGaAs nanoantenna, we calculate a conversion efficiency up to 10$^{-7}$ W$^{-1}$ at around 11 THz for an optimized structure. Interestingly above 10 THz there is a dramatic reduction of the atmospheric absorption^30^. Moreover, we also stress that the same approach can be applied also using different materials with second-order nonlinearity, such as LiNbO$_3$ to access different spectral regions.

These findings demonstrate that dielectric nanoantennas can represent powerful THz devices and of wide importance in material science and optical engineering sectors.

## Results

Let us imagine a scenario in which a dielectric metasurface generates a THz signal through DFG process when excited with pumps in the infrared regime, see Fig. 1. The optical-rectifying metasurface (OR-MTS) can be encapsulated at the center of a THz metasurface. In this context, the OR-MTS represents a THz launcher that has the scope to excite a THz surface wave on the THz-MTS. The latter, in its simpler version, is azimuthally symmetric around the OR-MTS and is constituted by a distribution of gold micropillars, very dense in terms of the THz wavelength; like a scaled version of the one presented in^11^. By properly optimizing the THz-MTS it is possible to generate a broadside radiation at THz by modulating the THz MTS through the heights of the nanopillars. To this end, the radial period of the modulation should match the wavelength of the surface wave excited at terahertz, thus providing an energy conversion into a leaky-mode. The first and fundamental step in this design, which also coincides with the main results of this paper, is the engineering of the single nanoresonator that directly converts light from Infra-Red (IR) to the THz with the highest efficiency of SW excitation. Moreover, let us stress that the dielectric antennas not only strongly boost the DFG mechanism due to the bulk nonlinearities, but also provides a suitable nonlinear radiation pattern for launching surface waves in THz metasurfaces.

To demonstrate this statement, we aim to exploit nonlinear DFG process in an all-dielectric nanoantenna of AlGaAs crystalline structure. Indeed, starting from two intense laser beams in the infra-red, the DFG nonlinear generated field can have a frequency of few THz, thus laying within the THz gap window. Although there exist other compounds that possess zincblende structure, the choice of AlGaAs is rather advantageous for our purpose, due to its low loss and dispersion in the optical region, high refractive index, large nonlinear susceptibility of the second-order $\chi ^{(2)}$, as well as a well-established fabrication techniques^23,31^.

**Figure 2:**
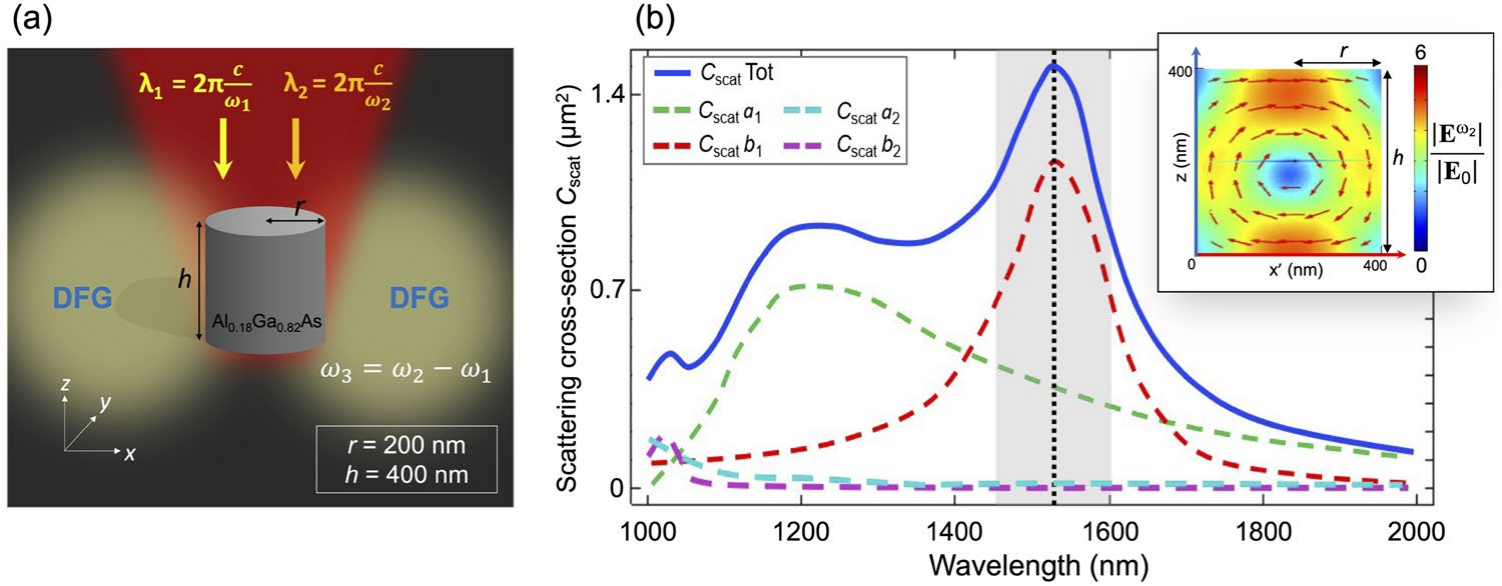
(**a**) The proposed AlGaAs nanodisk has radius *r* = 200 nm, and height *h* = 400 nm. Two plane waves in the near-infrared region, $\lambda _1$ and $\lambda _2$, are normally incident and generate through the bulk $\chi ^{(2)}$ the nonlinear DFG signal. The schematic representation elucidates the THz generation by optical rectification of intra-pulse components of the incident infrared laser pump. (**b**) Scattering cross section decomposed in the multipolar contributions: electric ($a_1$), magnetic ($b_1$) dipole and electric ($a_2$), magnetic ($b_2$) quadrupole components, respectively. The gray area indicates the wavelength of the input beams. The inset shows the electric field enhancement inside the nanodisk at the MD resonance (black dotted line), in a plane (zx′) forming $45^{\circ }$ with respect to the x and y axes. The red arrows highlight the circulating electric field. The plot is obtained by using COMSOL Multiphysics v5.5—https://www.comsol.com.

Let us consider an AlGaAs nanocylinder free-standing in air, with radius *r* equal to 200 nm and height *h* of 400 nm as shown in Fig. 2a. The geometrical parameters of the nanodisk are selected in order to fulfill a magnetic dipolar resonance around the fundamental wavelength ($\lambda _1$ and $\sim \lambda _2$), (see Fig. 2b for the complete multipolar decomposition^23^). We perform numerical simulations by means of the frequency domain analysis available in the Wave Optics module of the COMSOL Multiphysics software. We consider two incident pump beams that excite the proposed nanodisk in the infra-red range. The two incident signals, which logically correspond to the IR pump beam and information signal, are modeled as plane waves linearly polarized at $45^{\circ }$ (L45) with wavelength respectively equal to $\lambda _1$ and $\lambda _2$. In particular, we fix $\lambda _2$ to 1550 nm and we vary $\lambda _1$ from 1680 to 1550 nm to shift the information signal from the optical to the THz domain. Thus, two spectrally close components (having angular frequencies $\omega _1$ and $\omega _2$) of the input optical pulses are mixed via the DFG process, so that the terahertz component is generated. At this initial stage, we are neglecting the bandwidth of the information content around $\omega _2$.

**Figure 3:**
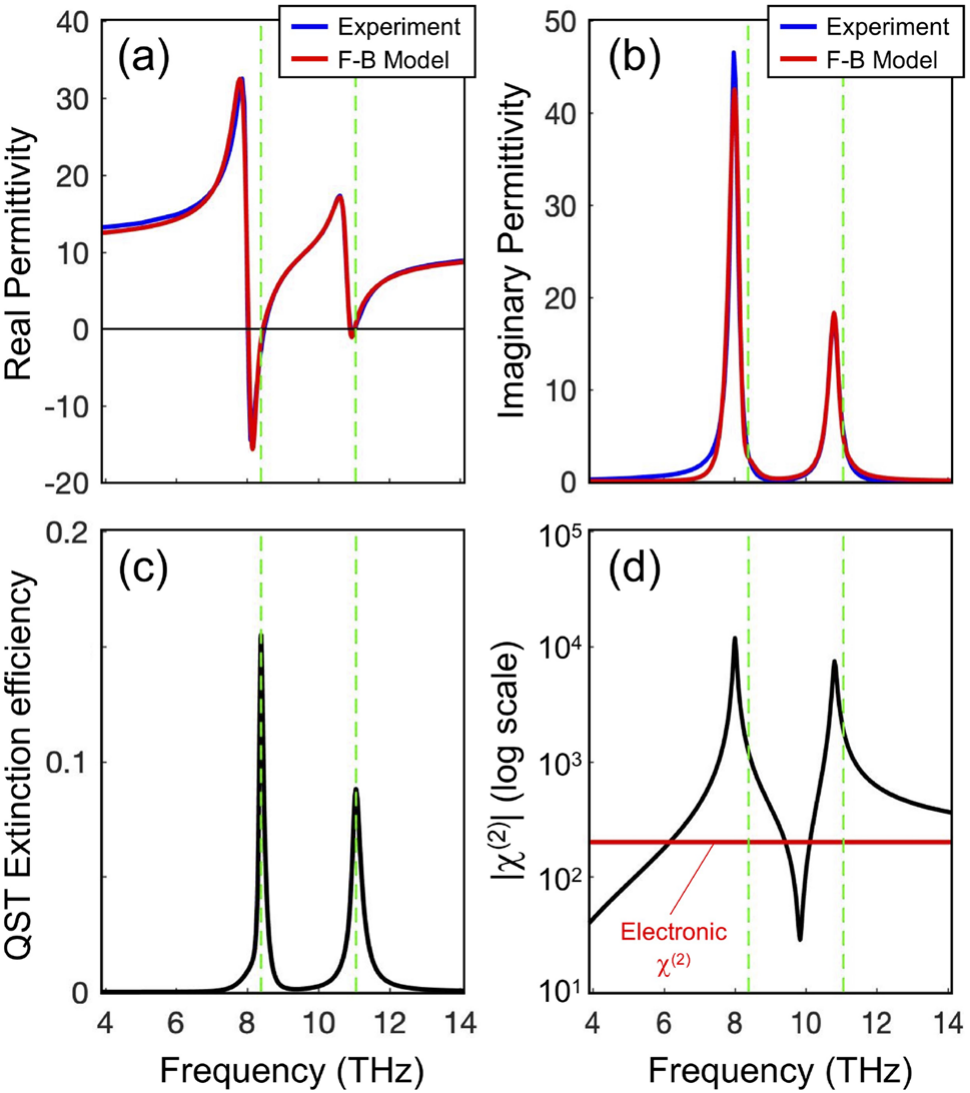
(**a**,**b**) Complex permittivity of AlGaAs in the THz region, showing the comparison between the experimental data (blue curve) and the F-B model (red curve) used in our simulation. (**c**) Linear extinction cross-section normalized to the pillar area estimated from quasi-static formulas. The resonance frequencies of the localized phonon-polaritons are marked by vertical green dashed lines. (**d**) Second-order susceptibility from Eq. (1). The $\chi ^{(2)}_E$ electronic contribution is also shown in red.

Apart from the linear solution of the fields at the pump optical frequencies $\omega _1$ and $\omega _2$, to calculate the nonlinear DFG signal at $\omega _3$ the knowledge of the linear material properties at the THz frequency is required. In order to consider a valid model for our simulations, we have implemented the so-called Forouhi-Bloomer (F-B) dispersion relations^32^. These are a set of equations for the complex refractive index $N(E) = n(E) - ik(E)$ of insulators and semiconductors as a function of photon energy *E*, which are valid over wide ranges of the EM spectrum, spanning from radio waves up to extreme UV. To get the dispersion curves for AlGaAs, we must use the few available data in literature to fit the model parameters, so that we are later able to make a broader extrapolation of *n* and *k* over the desired far-IR region. As suggested in^21^, the Levenberg-Marquardt method is used to fit the analytical model with the empirical data reported in^33,34^, in which a proper choice of the initial parameters is more than pertinent. The regression analysis is performed in Matlab and the corresponding dispersion curves are reported in Fig. 3, in terms of the complex permittivity $\varepsilon = N^2$. A good agreement between experimental data and F-B model is observed. Note that the permittivity spectrum is dominated by two resonances at around $\bar{\omega }_1/2\pi = 8.004$ THz and $\bar{\omega }_2/2\pi = 10.809$ THz, which are ascribable to the Transverse Optical (TO) phonon modes of AlAs and GaAs, respectively, in agreement with the estimation of AlGaAs permittivity for specific Al molar fractions reported in^35^. Note that around the TO phonon resonances a band of negative permittivity (real part) arises (Fig. 3a). This in turn enables the excitation of localized surface phonon-polaritons (SPhP)^36^, providing resonant enhancement of the linear extinction spectrum (black trace in Fig. 3c), here evaluated from quasi-static formulas for spherical scatterers^37^. Note that the two SPhP extinction peaks (marked by vertical green lines in Fig. 3) are detuned with respect to the TO phonon absorption peaks of bulk AlGaAs (cf. Fig. 3b), thus enabling the onset of high quality factor SPhP resonances at THz frequencies from nanoscale antennas.

To perform simulations of the DFG process in Comsol, the knowledge of the AlGaAs nonlinear susceptibility $\chi ^{(2)}$ in the THz region is mandatory. Far away from the material resonances, Miller’s (empirical) rule is an interesting tool for determining the second-order nonlinear susceptibility from the material linear properties. However, the presence of TO phonon resonances in the THz region, prevents the exploitation of Miller’s rule in our case. Instead, the so-called Faust-Henry model can be used^38^. According to this method, the nonzero component of the $\chi ^{(2)}$ tensor in zincblende structures reads as follows:

1 $$\begin{aligned} \chi ^{(2)}(\omega _3;\omega _2,-\omega _1) = \chi _E^{(2)}(\omega _{1,2}) \; + \sum _{p = 1}^{M} \frac{\bar{\omega }_p^2 C_p \chi _E^{(2)} (\omega _{1,2})}{ \bar{\omega }_p^2 + i\Gamma _p \omega _3 - \omega _3^2 }\,, \end{aligned}$$

where $\chi _E^{(2)}$ $(\omega _{1,2})$ is the purely electronic contribution at the near-IR frequencies, $\bar{\omega }_p$ is the TO phonon resonance frequency (above detailed), $\Gamma _p \sim 0.01 \bar{\omega _p}$ is the phenomenological damping factor and $C_p$ is the dimensionless Faust-Henry coefficient that measures the strength of the ionic contribution relative to the electronic one. AlGaAs shows a two-mode phonon behavior (i.e. *M* = 2), thus the following values have been implemented in our model: $\chi _E^{(2)}$ $(\omega _{1,2})$ = 200 pm/V, $C_1 = - 0.59$, and $C_2 = - 0.37$. The resulting $\chi ^{(2)}$ spectrum (in modulus) is shown in Fig. 3d (black trace). Note that, similarly to what observed for the linear permittivity, the second-order nonlinearity is also dominated by TO phononic features, boosting the $\chi ^{(2)}$ well above the non-resonant electronic contribution $\chi ^{(2)}_E$ (red trace in Fig. 3d) at around the TO phononic peaks ($\bar{\omega }_{1,2}$). The DFG process is thus expected to be strongly enhanced close to the TO peaks.

In the following, the key steps for the numerical implementation of the DFG process in Comsol Multiphysics are briefly described. The model consists of three computational steps: in the first two, the linear problem is solved at the pump frequencies $\omega _{1,2}$ and the results are used to calculate the nonlinear current sources. These first two steps are based on the scattering field formulation, where the background field is an IR L45-polarized light. The third one deals with the problem at the THz frequency $\omega _3 = \omega _2 - \omega _1$ and it involves a full-field simulation where the nonlinear currents imposed within the AlGaAs nanocylinder are the sources for the THz field, reading:

2 $$\begin{aligned} J_i(\omega _3) = -i\omega _32\varepsilon _0\chi _{ijk}^{(2)}(\omega _3;\omega _2,-\omega _1) (E_j(\omega _2)E_k^*(\omega _1)+E_k(\omega _2)E_j^*(\omega _1)) \end{aligned}$$

**Figure 4:**
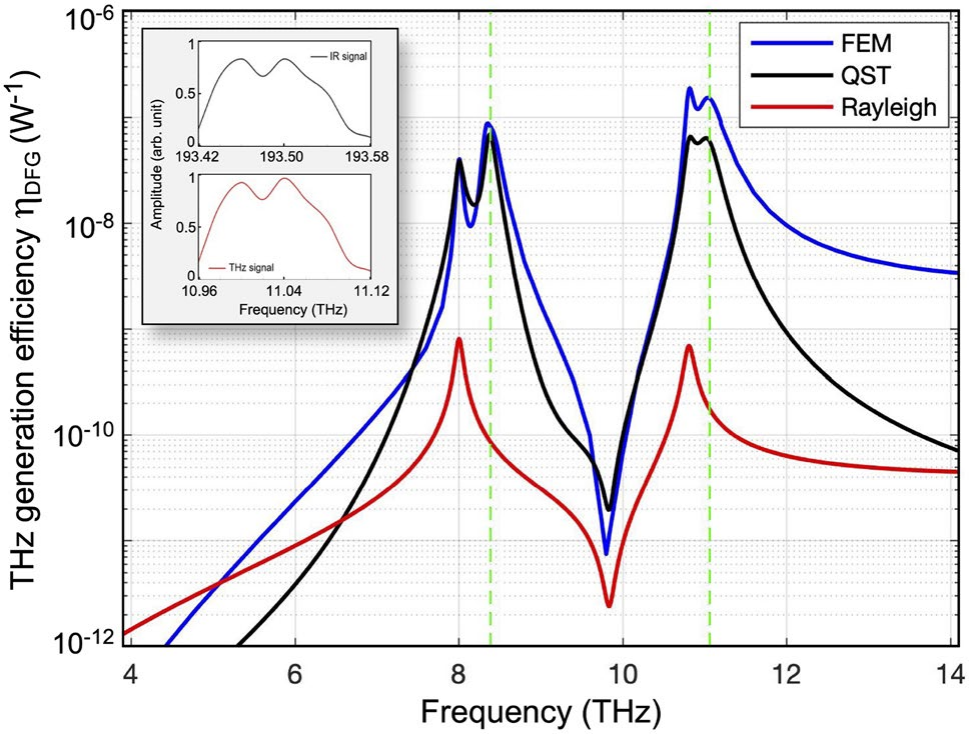
Conversion efficiency for THz generation by DFG in the proposed AlGaAs nanoantenna, evaluated from FEM numerical analysis (blue), and QST reduced model of Eq. (4) with (black) or without (red) contribution from TO phonon permittivity. The inset shows the negligible spectral distorsion introduced by the proposed transceiver.

$i\ne j\ne k$ being the Cartesian coordinates. A critical computational aspect of the DFG process under study is that it comprises physical problems with extremely different characteristic length scales. At the optical regime, the working wavelengths have dimensions of few μm, while the generated THz field at $\omega _3$ is in the range of tens to thousands $\upmu$m. Consequently, the size of the computational domain must be large enough to embody at least an entire wavelength at $\omega _3$ but, simultaneously, its discretization must be fine enough to accurately resolve the fundamental fields at $\omega _{1,2}$. To avoid an extremely high memory requirement for the model execution, we thus implement a geometry (and related mesh) based on two different components: one for the optical problems and a second one for the THz problem in which the domain is bigger. This allows us to significantly relax the minimum mesh element size in the host medium (air) when solving the THz simulation, but still maintain the same finer mesh as that at the near-IR inside the nanocylinder. In details, a General Extrusion coupling operator is used to connect the nanocylinders of both components; with this, the wave equation solutions at $\omega _{1,2}$ (NIR range) are mapped to an expression that can be evaluated in the destination geometry (THz range). Regarding DFG, one of the most important parameters in nonlinear optical processes is the conversion efficiency. In this sense, it is preferable to provide a result that is independent of the input power and that is characteristic of the nanoresonator itself^39^. Hence, we define the optical to THz efficiency as:

3 $$\begin{aligned} \eta _{DFG} = \frac{P_{rad}^{\omega _3}}{P_{i} \cdot P_s} = \frac{ \iint _\Sigma \vec {S}_{DFG} \cdot \vec {n} dS}{(I_0\pi r^2)^2}\,, \end{aligned}$$

where $P_{rad}^{\omega _3}$ is the power radiated at THz, $P_{i}$ and $P_s$ are the incident powers of the information signal and the pump beam, respectively; $\vec {S}_{DFG}$ is the Poynting vector of the DFG field, $\vec {n}$ is the unit vector normal to a surface $\Sigma$ enclosing the antenna, *r* is the pillar radius (200 nm). For simplicity, we are assuming that both the IR information and IR beam have the same incident intensity $I_0$ = 1 GW/cm$^2$. However, please note that since DFG is a second-order nonlinear process, our definition is independent from the input intensity. Thus, we compute the trend of the AlGaAs nanocylinder $\eta _{DFG}$ over a spectral range in the THz regime. To do so, we fix $\omega _2$ to 2$\pi$ (193.41 THz) and $\omega _1$ is swept across a band of values yielding a DFG frequency that spans from 4 to 12 THz as reported in Fig. 4. The numerical results indicate that $\eta _{DFG}$ basically follows the dispersion of $\chi _{ijk}^{(2)}(\omega _3;\omega _2,-\omega _1)$ (cf. Fig. 3d), even though not exactly. Actually, differently to the $\chi ^{(2)}$, conversion efficiency exhibits four peaks: two of them are located at the spectral position of the TO phonon frequencies $\bar{\omega }_{1,2}$, thus belonging to the bulk $\chi ^{(2)}$ spectral features, whereas another couple of peaks precisely sit at the SPhP resonances of the nanopillar (cf. Fig. 3c).

The role of SPhPs can be investigated by resorting to a reduced quasi-static model of the THz generation process. This is based on the assumption that the efficiency of the process is proportional to the quality factors $Q_1$ and $Q_2$ of the IR resonances interacting with the two pumps at $\omega _1$ and $\omega _2$, to the quasi-static linear extinction cross-section $\sigma _{ext}$ at $\omega _3$, and, of course, to the modulus of the $\chi ^{(2)}$. This way, one can estimate the DFG efficiency according to the following semi-analytical formula:

4 $$\begin{aligned} \eta _{DFG}^{QST}(\omega _3) = A Q_1 Q_2 \sigma _{ext}(\omega _3) |\chi ^{(2)}(\omega _3)|\,, \end{aligned}$$

where *A* is a fitting parameter expressed in W$^{-1}$m$^{-2}$/(pm/V). The estimation provided by Eq. (4) is reported in Fig. 4 (black trace), assuming $Q_1 = Q_2 \simeq 12$ from Fig. 2b, $\sigma _{ext}$ taken from Fig. 3c, and by setting $A = 20$. A fair qualitative agreement with the extinction efficiency evaluated from FEM numerical analysis (blue trace in Fig. 4) is retrieved. Note that if, instead of the SPhP extinction spectrum of Fig. 3c, one considers non-resonant extinction from the Rayleigh background, the estimated DFG efficiency would drop by almost two orders of magnitude on the efficiency peaks (red curve in Fig. 4). This ascertains the key role of SPhP in the THz generation from all-dielectric nonlinear nanoantennas.

**Figure 5:**
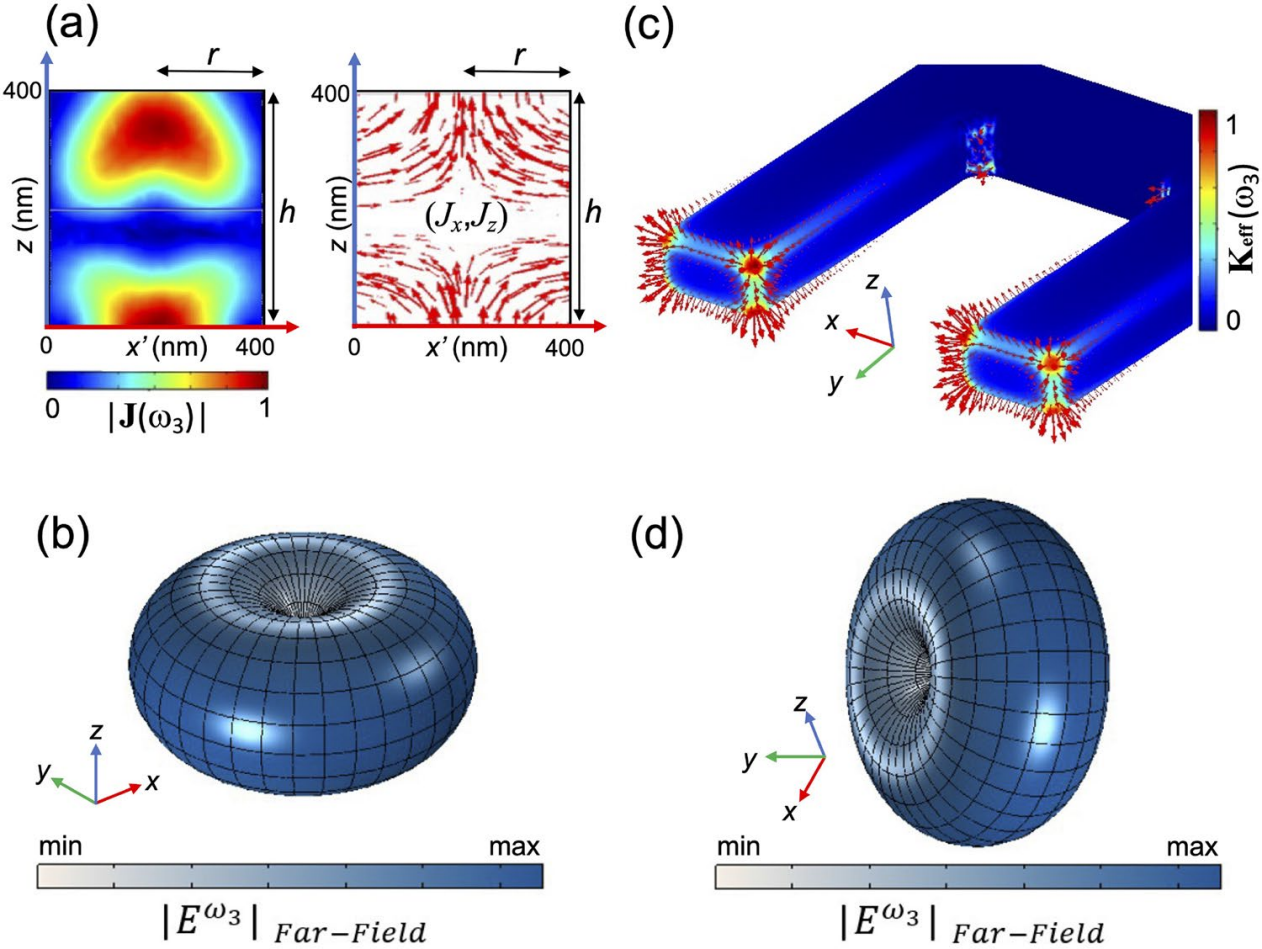
(**a**) The spatial distribution of the normalized nonlinear currents inside the nanocylinder at $\omega _{3}$: modulus (left panel) and orientation (right panel). (**b**) Far-field emission of the AlGaAs nanoantenna in the THz. (**c**) The normalized effective surface currents in the gold SRR at $\omega _{3}$. (**d**) Radiation pattern of the SRR in the THz. The plots are obtained by using COMSOL Multiphysics v5.5—https://www.comsol.com.

Most interestingly, note that the peak of $\eta _{DFG}$ achieved at 10.81 THz is as high as $\sim 2\times 10^{-7}$ W$^{-1}$, and this THz radiation is generated by a dipolar nonlinear source aligned with the vertical axis of the nanocylinder. To elucidate this issue, Fig. 5a shows the spatial distribution of the nonlinear currents inside the AlGaAs nanoantenna at $\omega _3$ equal to 2$\pi$ (10.81 THz). As can be noticed, the *z*-component of the nonlinear current is equally directed both in the upper half and lower half of the nanocylinder. Nonetheless, the *x*- and *y*-components (much weaker than the *z* one) are oppositely directed in each half region and so we expect their far-field contributions to cancel out. This is also confirmed by analyzing the THz far-field emission directly from the simulation, see Fig. 5b: the AlGaAs nanocylinder effectively behaves as an electric point dipole antenna and its dipole moment is oriented along the nanocylinder axis. We consider also the more realistic case in which the information message has a specific bandwidth, B, around 1550 nm. For instance, by assuming B = 160 GHz, the inset of Fig. 4 proves that the entire information bandwidth can be converted around 11 THz with negligible spectral distortion.

Lastly, to further highlight the potential of the proposed structure for THz applications, we perform a comparison with state-of-the-art configurations, based on a plasmonic structure. We consider an isolated gold split-ring resonator with similar geometrical parameters as the one reported in^22^, which allows to excite a localized plasmonic resonance at a pump wavelength of around 1.5 μm. The second-order nonlinearity of such metallic nanoparticles preserves a surface nature and the predominant component of the nonlinear current density points towards the normal direction of the nanoantenna corners, see Fig. 5c. For the surface $\chi ^{(2)}$ value of gold, we adopted the well established hydrodynamic model for second harmonic generation (SHG) available in literature^40,41^, yielding a value of $|\chi ^{(2)S}_{\bot \bot \bot } |\simeq 4 \cdot 10^{-18} \text{ m}^{2}/\text{V }$. Similarly, this sheet nonlinear susceptibility enabled us to reproduce the experimental results of THz generation reported in^22^ at around 2 THz, by imposing the aforementioned surface nonlinear currents as THz sources of the gold SRR. Such THz generation efficiency simulations retrieve $\eta _{DFG}$ values of the order of 10^−15^ W^−1^ around 8 THz and of 10^−14^ W^−1^ around 10 THz, which respectively are 8 and 7 orders of magnitude lower than what is attained with the proposed single AlGaAs nanopillars. Moreover, as shown in Fig. 5d, this emission is mostly out-of-plane because of the different nature of the resonances exploited in the split-ring configuration (see “Methods” section for the details). Hence, using high-refractive index dielectric materials not only strongly enhances the DFG process thanks to the bulk $\chi ^{(2)}$ and THz SPhP oscillations, but also provides, from a simple cylindrical geometry of the antenna, a configuration of the nonlinear radiation pattern that is suitable for launching surface waves in THz metasurfaces.

## Discussion

In this work, we demonstrate a novel method for the direct conversion from IR to THz regime at the nanoscale. Both the IR carrier and information signal which are spectrally close to each other are used to excite a dielectric nanoantenna of AlGaAs. Through the DFG mechanism of the input beams, the IR information light is converted into a signal that lays in the THz region. We reveal a record high value of THz efficiency up to 10$^{-7}$ W$^{-1}$ from a single AlGaAs nanocylinder, which is an improvement of about 8 orders of magnitude compared to state-of-the-art nanoscale THz generation, based on gold nanoresonators, even if the material is lossy in the THz region. This result stems from the following key advantages offered by the AlGaAs nanoantenna platform: (i) second-order nonlinear processes in AlGaAs arises from bulk instead of surface effects, typical of centro-symmetric noble metal nanostructures; (ii) the bulk $\chi ^{(2)}$ of AlGaAs is boosted by TO phonons; (iii) the phononic linear permittivity enables surface phonon-polariton resonances, at around 8.5 and 11 THz, in AlGaAs nanostructures. Moreover, the nanopillar configuration is capable of efficient in-plane emission of the nonlinear generated signal, when pumped at normal incidence, which is beneficial in view of an integration into large area metasurfaces for simultaneous encoding and beam steering of free-space THz radiation. Our results can pave the way to the development of ultracompact THz transmitter/receiver fully transparent to the signal modulation format for next generation THz photonics.

## Methods

### Nonlinear AlGaAs susceptibility at THz

At the THz regime, the bulk $\chi ^{(2)}_{xyz}$ AlGaAs nanocylinder is described by the so-called Faust-Henry model:

5 $$\begin{aligned} \chi ^{(2)}_{xyz}(\omega _{3},\omega _{2},-\omega _{1}) = \chi ^{E(2)}_{xyz}(\omega _{1,2}) + \sum ^{N}_{p = 1} C_{p}\chi ^{E(2)}_{xyz}(\omega _{1,2})\frac{\omega _{p}^{2}}{\omega _{p}^{2} - \omega ^{2}_{3} + i\gamma _{p}\omega _{3}}\,, \end{aligned}$$

where $\chi ^{E(2)}_{xyz}(\omega _{1,2})$ is the purely electronic contribution at the near-IR frequencies, *N* is the number of TO resonances in the far-IR region, $\omega _{p}$ is the TO phonon resonance frequency, $\gamma _{p} \simeq 0.01\omega _{p}$ is the phenomenological phonon damping factor and $C_{p}$ is the dimensionless Faust-Henry coefficient that measures the strength of the ionic contribution relative to the electronic one^42^. Therefore, $\chi ^{(2)}$ is now expressed as the sum of the conventional IR electronic term (which, below electronic transitions, is real and constant) and a sum of Lorentz oscillators, accounting for the lattice resonances. The Faust-Henry model can be extended to more generic second-order nonlinear interactions in zincblende structures as follows:

6 $$\begin{aligned} \begin{aligned} \chi ^{(2)}(\omega _{1}+\omega _{2},\omega _{1},\omega _{2})&= \chi ^{(2)}_{\infty } \bigg [ 1 + C_{1}\left( \frac{\omega ^{2}_{TO}}{D(\omega _{1})} + \frac{\omega ^{2}_{TO}}{D(\omega _{2})} + \frac{\omega ^{2}_{TO}}{D(\omega _{1} + \omega _{2})} \right)  \\ & \quad + C_{2} \left( \frac{\omega ^{2}_{TO}}{D(\omega _{1})D(\omega _{2})} + \frac{\omega ^{2}_{TO}}{D(\omega _{2})D(\omega _{1} + \omega _{2})} \bigg . + \frac{\omega ^{2}_{TO}}{D(\omega _{1} + \omega _{2})D(\omega _{1})}\right)  \\ & \quad+ C_{3}\left( \frac{\omega ^{2}_{TO}}{D(\omega _{1})D(\omega _{2})D(\omega _{1}+\omega _{2})} \right) \bigg ]\,, \end{aligned} \end{aligned}$$

where $D(\omega ) = \omega ^{2}_{TO} - \omega ^{2} - i\gamma \omega$. The parameters of the Faust-Henry model in Eq. (5) must be determined either by experiments or theoretical calculations. The values that have been used in this work are^43,44^:

7 $$\begin{aligned} &\chi ^{E(2)}_{xyz}(\omega _{1,2}) = 200 \text{ pm/V } \,, N = 2 \,, C_{1} = -0.59 \,, \omega _{1} = 8.004 \text{ THz } \text{(GaAs-like } \omega _{TO}) \,,\\ & C_{2} = -0.37 \,, \omega _{2} = 10.809 \text{ THz } \text{(AlAs-like } \omega _{TO})\,. \end{aligned} $$

**Figure 6:**
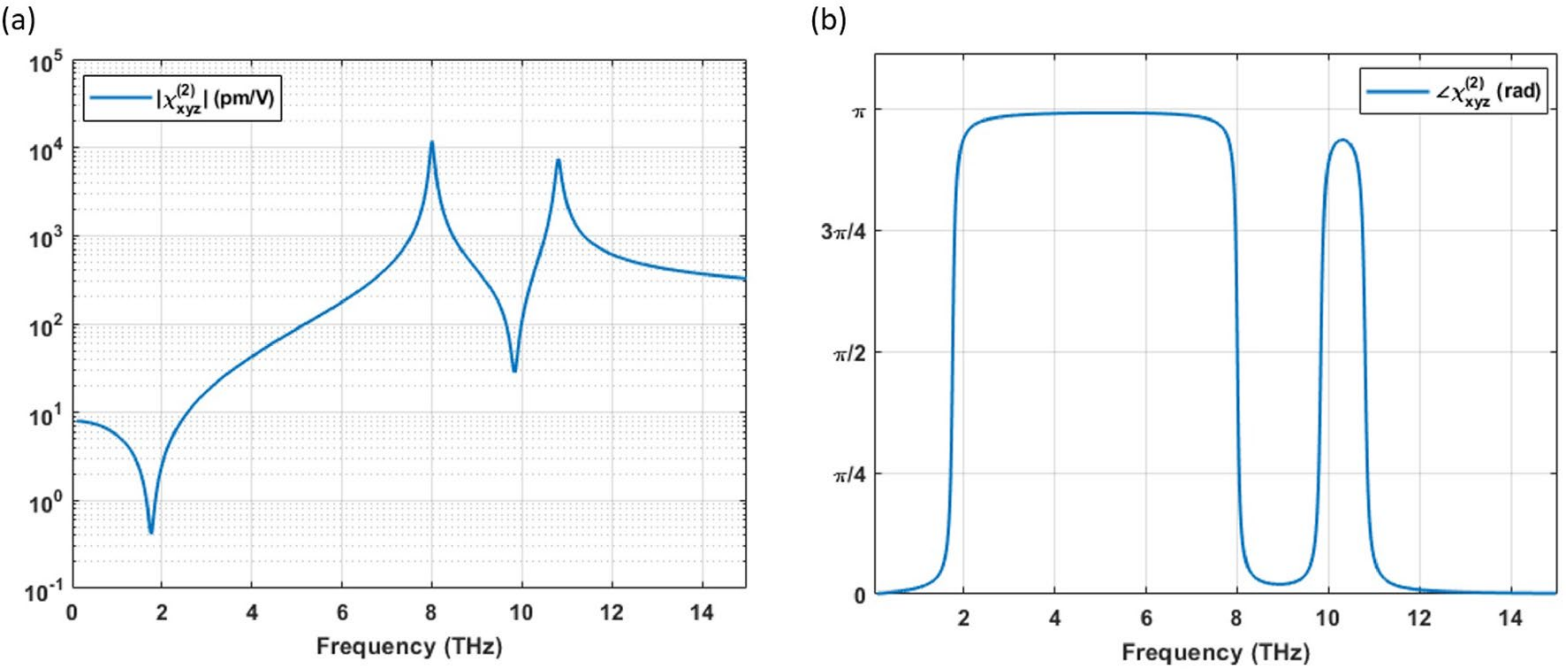
Dispersion of $\chi ^{(2)}_{xyz}(\omega _3,\omega _2,-\omega _1)$ of AlGaAs in the THz region: (**a**) magnitude and (**b**) phase.

The negative values of $C_{1}$ and $C_{2}$ reveal the phase difference between the ionic and electronic contributions. The resulting dispersion of AlGaAs’s $\chi ^{(2)}_{xyz}$ in the THz region is shown in Fig. 6.

### Comparison with plasmonics nanoresonators

**Figure 7:**
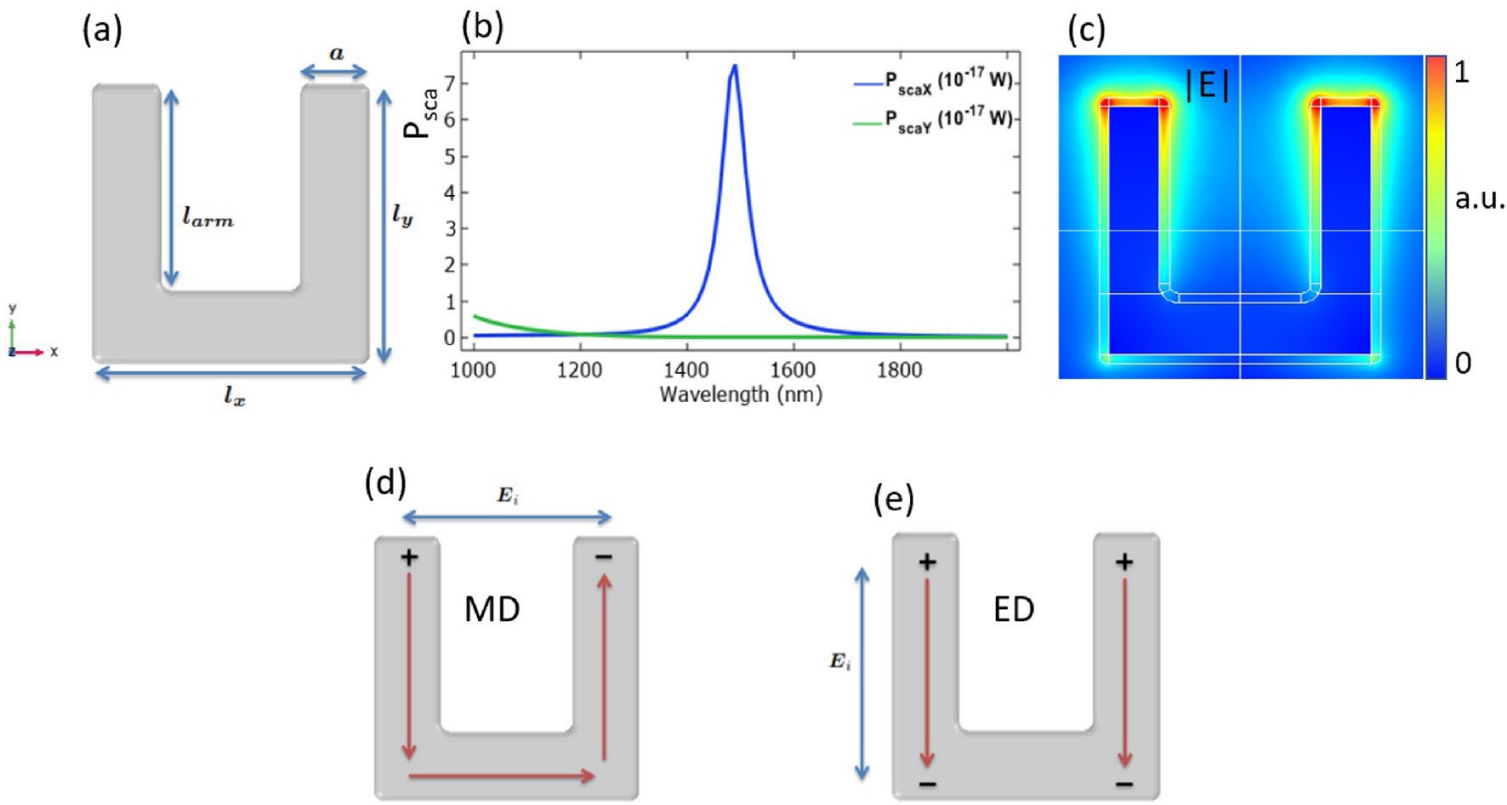
Sketch of the proposed air-surrounded gold SRR and (**b**) its scattering spectrum. (**c**) The electric field norm around $\lambda$ = 1.5 $\upmu$m reveals the LSPR. The corners and edges of the metallic structure are smoothed by a curvature of 5 nm to avoid numerical singularities. The plot is obtained by using COMSOL Multiphysics v5.5—https://www.comsol.com. (**d**) Schematics of the linear current distributions at the magnetic dipole, MD, and (**e**) electric dipole, ED, resonances of the SRR.

The direct competitors of high refractive index dielectric nanoantennas are metallic nanoresonators, which have been extensively investigated. The main goal of this section is to get an estimation of the DFG conversion efficiency in an isolated metallic nanostructure. The so-called split-ring resonator (SRR) is one of the most common metallic nanoelement since it provides high nonlinear conversion efficiencies and it is usually employed as the building block of more sophisticated metamaterials. Let us consider a SRR made of gold with geometrical parameters as in^45^ that guarantee a resonant behaviour in the optical regime. As can be observed in Fig.7 the nanostructure is resonant around $\lambda$ $\sim$ 1.5 $\upmu$m for a pump excitation linearly polarized along the *x*-axis: this resonance corresponds to a localized surface plasmon (LSP) mode, and, as can be appreciated in Fig. 7c, the electric field is strongly localized at the corners of the gold SRR. In more details, the *x*-polarized pump beam induces a circular current flow along the length of the SRR, leading to a strong magnetic dipole moment, (Fig.7d). This is why the LSP resonance at $\lambda$ $\sim$ 1.5 μm is also called the fundamental magnetic dipole. Instead, for *y*-polarized incident light the so called electric dipole mode is excited. However, the latter is located at wavelengths below the considered IR window. The selected metallic nanoparticle is linearly resonant at the third communication window and this makes the gold SRR a good candidate for a fair comparison with the AlGaAs nanocylinder presented in the main text.

Following the hydrodynamic model it is possible to derive the expression of the nonlinear current densities in the undepleted pump approximation. For the second harmonic generation (SHG) process, assuming a single pump field with frequency $\omega _1$ incident on the metallic nano-object, the current density oscillating at $\omega _2=2\omega _1$ reads as:

8 $$\begin{aligned} \begin{aligned} \varvec{J}(\varvec{r},\omega _{2})&= \frac{e^{2}n_{0}}{m(-i\omega _{2}+\gamma _{0})}\varvec{E}(\varvec{r},\omega _{2}) + \varvec{J}^{S}_{NL}(\varvec{r},\omega _{2}) = \frac{e^{2}n_{0}}{m(-i\omega _{2}+\gamma _{0})}\varvec{E}(\varvec{r},\omega _{2})  \\ & \quad+ \frac{\varvec{J}(\varvec{r},\omega _{1})}{n_{0}e(-i\omega _{2}+\gamma _{0})}\left( \nabla \cdot \varvec{J}(\varvec{r},\omega _{1}) + \varvec{J}(\varvec{r},\omega _{1}) \cdot \nabla \right) + \frac{ie}{m\omega _{1}}\frac{\nabla \cdot \varvec{J}(\varvec{r},\omega _{1})}{(-i\omega _{2}+\gamma _{0})}\varvec{E}(\varvec{r},\omega _{1})\,, \end{aligned} \end{aligned}$$

where only surface (S) nonlinear contributions have been conceived in the derivation of $J(r,\omega _2)$. Instead, for the DFG, the nonlinear interaction of the free electrons with the two input pump fields at $\omega _1$ and $\omega _2$ yields the following oscillating current density at $\omega _3=\omega _2-\omega _1$:

9 $$\begin{aligned} \begin{aligned} \varvec{J}(\varvec{r},\omega _{3}) =&\frac{e^{2}n_{0}}{m(-i\omega _{3}+\gamma _{0})}\varvec{E}(\varvec{r},\omega _{3}) + \varvec{J}^{S}_{NL}(\varvec{r},\omega _{3}) = \frac{e^{2}n_{0}}{m(-i\omega _{3}+\gamma _{0})}\varvec{E}(\varvec{r},\omega _{3}) \\ & \quad + \frac{1}{n_{0}e(-i\omega _{3}+\gamma _{0})}\left( \varvec{J}(\varvec{r},\omega _{2}) \left[ \nabla \cdot \varvec{J}^{*}(\varvec{r},\omega _{1})\right] + \left[ \varvec{J}(\varvec{r},\omega _{2}) \cdot \nabla \right] \varvec{J}^{*}(\varvec{r},\omega _{1}) \right) \\ & \quad + \frac{ie}{m\omega _{2}}\frac{\nabla \cdot \varvec{J}(\varvec{r},\omega _{2})}{(-i\omega _{3}+\gamma _{0})}\varvec{E}^{*}(\varvec{r},\omega _{1}) + \left( 2 \leftrightarrow 1^{*}\right) \,, \end{aligned} \end{aligned}$$

where $2 \leftrightarrow 1^{*}$ denotes the interchange between the quantities at $\omega _{2}$ and the complex conjugated ones at $\omega _{1}$. As in SHG, the response at the fundamental frequencies is taken to be exclusively linear. In the previous equations *e* is the absolute value of the electron charge, $n_0$ the equilibrium free electron density in the absence of applied fields, $\gamma _0$ the damping and *m* is the effective electron mass. We perform Comsol Multiphysics numerical calculation of these two nonlinear process. As already reported in the manuscript, once the wave equation is numerically solved for the pump fields and the material parameters are known, the nonlinear source can be constructed in a subsequent step and the desired harmonic field can be obtained from the corresponding full-field simulation. An useful approach to numerically imposing nonlinear volume current densities is to calculate what is known as the effective surface current density^46^:

10 $$\begin{aligned} \varvec{K}_{eff}(\varvec{r},\omega ) = \int \varvec{J}^{S}_{NL}(\varvec{r},\omega ) d\xi \,, \end{aligned}$$

where $\xi$ is the spatial coordinate along the direction normal to the metal boundaries and the integration is performed over the selvedge region (i.e. few angstroms from the surface). Note that $\varvec{K}_{eff}$ has dimensions of A/m. This approach is valid since $\varvec{J}^{S}_{NL}$ is concentrated near the nanoparticle surface, and so it can be integrated and replaced by an effective nonlinear current sheet located at the metal-dielectric interface. The effective SH surface current calculated in^45^ is written as a local function of the fundamental fields evaluated inside the metallic surface:

11 $$\begin{aligned} \varvec{K}_{eff}(\varvec{r},2\omega _{1}) = \frac{i\omega _{1}}{n_{0}e}\left( P_{\bot }(\varvec{r},\omega _{1})P_{\parallel }(\varvec{r},\omega _{1}) \varvec{\hat{t}} + \frac{1}{2}\frac{3\omega _{1} + i\gamma _{0}}{2\omega _{1} + i\gamma _{0}}P^{2}_{\bot }(\varvec{r},\omega _{1}) \varvec{\hat{n}} \right) \,, \end{aligned}$$

where $P_{i}(\varvec{r},\omega _{1}) = \frac{J_{i}(\varvec{r},\omega _{1})}{-i\omega _{1}} = \frac{e^{2}n_{0}}{-i\omega _{1} m(-i\omega _{1}+\gamma _{0})}E_{i}(\varvec{r},\omega _{1})$ is the linear polarization at the fundamental frequency, and $\varvec{\hat{n}}$ and $\varvec{\hat{t}}$ are the local unit vectors normal ($\bot$) and tangential ($\parallel$) to the metal surface, respectively. This analytical model leads to SHG conversion efficiencies that are in agreement with published experimental values^45^, and so it represents a good benchmark for testing the validity of our numerical nonlinear plasmonic simulations in *Comsol*. For simplicity, only the normal component of $\varvec{K}_{eff}$ will be considered in the following, which has been observed to be the predominant nonlinear surface contribution^47^. The first simulation step of SHG has consisted on impinging an *x*-polarized plane wave at $\lambda _{1} = 1.5 \; \upmu$m, in order to excite the MD resonance at the FF. It is important to remark that metallic nanostructures possess a lower damage threshold than dielectric ones; therefore, an input power of 16 MW/cm^2^ (typical in experiments) has been used, far below the 1 GW/cm^2^ considered in the paper.

This has enabled us to accurately construct the nonlinear surface current density normal to the SRR surface, Fig. 5c. The nonlinear currents oscillate in-phase along each of the two SRR arms and their contributions along the *y*-axis (i.e. orthogonal to the SRR gap) interfere constructively in the far-field, yielding a radiation pattern characteristic of a *y*-directed electric dipole at the SH frequency, Fig. 5d. The integration of the scattered power density over the internal PML boundary yields $P^{2\omega _{1}}_{rad} \simeq 1.5 \cdot 10^{-12}$ W; this is in agreement with the values reported in^48^, where the same model for $\varvec{\hat{n}}\cdot \varvec{K}_{eff}$ is used. Concerning the conversion efficiency, we obtain a value of $\left( \eta _{SHG}\right) _{SRR} \simeq 2.8 \cdot 10^{-7}$ W^−1^. Hence, it is clearly observed that the followed method for implementing in *Comsol* the nonlinear sources giving rise to harmonic generation in plasmonic gold SRRs works well. Knowing this, let us now turn into the DFG process, which is the one we are most interested in. In this case, it is the nonlinear current $\varvec{J}_{NL}(\varvec{r},\omega _{3})$ in Eq. (9) that comprises the surface sources of the field at $\omega _{3} = \omega _{2} - \omega _{1}$.

For the DFG process, due to the limitations of the purely classical hydrodynamic model, it is desirable to handle with experimental results. In this sense, the work by Mai Tal and co-workers^22^ is taken as a benchmark. In^22^ they measured THz pulses generated by optical rectification in a plasmonic nonlinear metasurface, consisting of a two-dimensional periodic arrangement (1 mm $\times$ 1 mm) of gold SRRs with geometrical parameters similar to the ones reported in^45^. The designed metasurface is pumped by femtosecond laser pulses centered at $\lambda \simeq 1.5$ $\upmu$m, with an incident peak intensity of around 16 MW/cm^2^, and the number of illuminated SRRs is of the order of $N \simeq 10^{3}\times 10^{3} = 10^{6}$. The achieved total $P^{THz}_{sca}/P_{in}$ ratio is almost $10^{-9}$ around $\omega _{3} = 2\pi \cdot (1.5$ THz). Assuming that the periodicity is large enough to avoid coupling between SRRs, the total scattered power can be approximated by $P^{THz}_{sca} \simeq N^{2}\left( P^{\omega _{3}}_{sca}\right) _{SRR}$, where $\left( P^{\omega _{3}}_{sca}\right) _{SRR}$ is the power scattered by a single SRR. Hence, for the employed input power level, a total $P^{THz}_{sca}/P_{in} \simeq 10^{-9}$ corresponds to a $\left( P^{\omega _{3}}_{sca}\right) _{SRR} \simeq 10^{-22}$ W power scattered by each individual SRR.

This estimation is useful to numerically evaluate the DFG efficiency attained in the THz region by a single gold SRR. To do so, the numerical model previously validated for the SHG in the SRR is used. The effective nonlinear surface current numerically imposed as a *Weak Contribution* on the metallic surface is now parameterized as follows:

12 $$\begin{aligned} \varvec{\hat{n}} \cdot \varvec{K}_{eff}(\varvec{r},\omega _{3}=\omega _{2}-\omega _{1}) = -i\omega _{3}2\varepsilon _{0}\chi ^{(2)S}_{\bot \bot \bot }(\omega _{3},\omega _{2},-\omega _{1})E_{\bot }(\varvec{r},\omega _{2})E_{\bot }^{*}(\varvec{r},\omega _{1})\,, \end{aligned}$$

where $\chi ^{(2)S}_{\bot \bot \bot }$ is the effective surface nonlinear susceptibility of the SRR (with dimensions of m^2^/V). Several estimations can be made for deriving such a quantity based on the $\varvec{J}_{NL}(\varvec{r},\omega _{3})$ given by the hydrodynamic model. However, we take advantage of the experimental OR results to determine a good estimation for $\chi ^{(2)S}_{\bot \bot \bot }$. As a first approximation, the aforementioned results obtained for SHG at $\lambda _{1} = 1.5 \; \upmu$m in the gold SRR can be helpful. In fact, the $\varvec{\hat{n}}\cdot \varvec{K}_{eff}(\varvec{r},2\omega _{1})$ SH source can be rewritten from Eq. (11) as:

13 $$\begin{aligned} \varvec{\hat{n}}\cdot \varvec{K}_{eff}(\varvec{r},2\omega _{1}) = \frac{i\omega _{1}}{n_{0}e}\frac{1}{2}\frac{3\omega _{1} + i\gamma _{0}}{2\omega _{1} + i\gamma _{0}}\left( \varepsilon _{0}\left( \varepsilon _{1}(\omega _{1}) - 1 \right) \right) ^{2} E^{2}_{\bot }(\varvec{r},\omega _{1}) \equiv -i2\omega _{1}\varepsilon _{0}\chi ^{(2)S}_{\bot \bot \bot }(2\omega _{1},\omega _{1},\omega _{1})E^{2}_{\bot }(\varvec{r},\omega _{1})\, \end{aligned}$$

which leads to an effective surface nonlinear susceptibility of magnitude:

14 $$\begin{aligned} |\chi ^{(2)S}_{\bot \bot \bot }(2\omega _{1},\omega _{1},\omega _{1})|= \frac{\varepsilon _{0}}{n_{0}e}\frac{1}{4}\frac{|3\omega _{1} + i\gamma _{0} |}{|2\omega _{1} + i\gamma _{0} |} \left( \varepsilon _{1}(\omega _{1}) - 1 \right) ^{2} \simeq 4 \cdot 10^{-18} \text{ m}^{2}/\text{V }\,. \end{aligned}$$

Using the pump solutions at $\omega _{2} = 2\pi \cdot (200$) THz [$\lambda _{1} = 1.5$ $\upmu$m] and $\omega _{1} = 2\pi \cdot (198$ THz) [$\lambda _{1} = 1.515$ $\upmu$m], and setting $|\chi ^{(2)S}_{\bot \bot \bot }(\omega _{3},\omega _{2},-\omega _{1}) |= 4 \cdot 10^{-18} \text{ m}^{2}/\text{V }$, the surface current sheet in Eq. (12) imposed in the THz component of the *Comsol* DFG model yields $P^{\omega _{3}}_{rad} \simeq 4.6 \cdot 10^{-22}$ W, at $\omega _{3} = \omega _{2} - \omega _{1} = 2$ THz. Interestingly, such a scattered power level lays within the scale expected from the experimental results described above. As a consequence, it can be considered to be a good approach so as to have a sight at the optical-to-THz conversion efficiency that can be obtained by a single air-surrounded gold SRR. In this way, for the DFG process, the first pump field has been fixed at $\omega _{2} = 2\pi \cdot 200$ THz, while $\omega _{1}$ has been varied with a step of 2 THz, obtaining the following results:

$$\begin{aligned} &\omega _{1} = 2\pi \cdot (198 \text{ THz), } \quad \omega _{3} = 2\pi \cdot 2 \text{ THz } \quad \longrightarrow \quad \left( \eta _{DFG}\right) _{SRR} \simeq 9 \cdot 10^{-17} \text{ W}^{-1} \\&\omega _{1} = 2\pi \cdot (196 \text{ THz), } \quad \omega _{3} = 2\pi \cdot 4 \text{ THz } \quad \longrightarrow \quad \left( \eta _{DFG}\right) _{SRR} \simeq 1.03 \cdot 10^{-15} \text{ W}^{-1} \\&\omega _{1} = 2\pi \cdot (194 \text{ THz), } \quad \omega _{3} = 2\pi \cdot 6 \text{ THz } \quad \longrightarrow \quad \left( \eta _{DFG}\right) _{SRR} \simeq 3.95 \cdot 10^{-15} \text{ W}^{-1} \\&\omega _{1} = 2\pi \cdot (192 \text{ THz), } \quad \omega _{3} = 2\pi \cdot 8 \text{ THz } \quad \longrightarrow \quad \left( \eta _{DFG}\right) _{SRR} \simeq 9.82 \cdot 10^{-15} \text{ W}^{-1} \\&\omega _{1} = 2\pi \cdot (190 \text{ THz), } \quad \omega _{3} = 2\pi \cdot 10 \text{ THz }  \longrightarrow  \left( \eta _{DFG}\right) _{SRR} \simeq 1.84 \cdot 10^{-14} \text{ W}^{-1} \end{aligned} $$

Regarding the optical-to-THz conversion efficiency, if the values reported in the main text from the AlGaAs nanopillar are recalled, it can be stated that several orders of magnitude enhancement are obtained in the dielectric case with respect to that of the single gold SRR. In the THz region lying above $f_{3} \simeq 4$ THz, $\left( \eta _{DFG}\right) _{AlGaAs}$ is between 2 and 8 orders of magnitude above $\left( \eta _{DFG}\right) _{SRR}$. Concerning the region below 4 THz, the destructive interference between the ionic and electronic contribution to the AlGaAs $\chi ^{(2)}(\omega _{3},\omega _{2},-\omega _{1})$ makes the DFG efficiency in the nanocylinder comparable to the plasmonic SRR counterpart. However, in such a spectral region, the higher damage threshold of dielectric nanostructures can be exploited to obtain more output power, by using a more intense pump field (even if the efficiency remains the same). Ergo the reported numerical results suggest that the modeled AlGaAs nanoantenna might represent a valuable optical nonlinear nanostructure capable of enriching the terahertz gap of the EM spectrum.

## Data Availability

The datasets used and/or analyzed during the current study are available from the corresponding author on reasonable request.
